# Supplementary material for: Systematic Review on Working Mechanisms of Signaling Pathways in Fibrosis During Shockwave Therapy
Source: Int J Mol Sci. 2024 Oct 31;25(21):11729. doi: 10.3390/ijms252111729 (PMC11546838; doi:10.3390/ijms252111729)
Supplement: Supplementary file 1 [file ijms-25-11729-s001.zip › ijms-3274917-supplementary.pdf]

Supplementary Materials
S1: detailed overwiew of the search strategy:
key words:
PATIENT:
fibrosis, Pulmonary fibrosis, Oral submucous fibrosis, Endomyocardial fibrosis, Cystic fibrosis
tissue, connective tissue cells, connective tissue
skin, cutanous tissue, subcutanous tissue

INTERVENTION
extracorporeal shockwave therapy, shock Wave therapy, extracorporeal Shock Wave therapy, extracorporeal shockwave, Shockwave therapy, extracorporeal shockwave
Lithotripsy, extracorporeal shockwave lithotripsy, extracorporeal shockwave lithotripsies
Extracorporeal shockwave treatment, extracorporeal shock wave treatment,
Shockwave treatment, Shock wave treatment, Shockwave
High-energy shock waves, Shockwave, Radial pressure wave therapy, Radial pressure wave therapy
Acoustic wave therapy
ESWT, SWT, SW, RPWT, AWT, ESWT, SWT, SW, RPWT, AWT

OUTCOME
mechanotransduction/ cellular, target cell, signaling pathways, singal transduction, cellular, cells, cell physiology, eipithelial mesenchymal transition, micro RNA, exosomes

Search strategy:
Pubmed

**(Fibrosis[MeSH Terms] OR Fibrosis [Tiab] OR pulmonary fibrosis[MeSH Terms] OR Pulmonary fibrosis [Tiab] OR oral submucous fibrosis[MeSH Terms] OR oral submucous fibrosis[Tiab] OR endomyocardial fibrosis[MeSH Terms] OR endomyocardial fibrosis[Tiab] OR Cystic fibrosis[MeSH Terms] OR cystic fibrosis[Tiab] OR connective tissue cells[MeSH Terms] OR connective tissue cells[Tiab] OR skin[MeSH Terms] OR cutaneous tissue[Tiab] OR skin[Tiab] OR subcutaneous tissue[MeSH Terms] OR subcutaneous tissue[Tiab] OR connective tissue[MeSH Terms] OR connective tissue[Tiab] OR fibroblasts[MeSH Terms] OR fibroblasts[Tiab] OR myofibroblasts[MeSH Terms] OR myofibroblasts[Tiab] OR tissue[Tiab] OR tissues[MeSH Terms]) AND ("extracorporeal shockwave therapy"[MeSH Terms] OR extracorporeal shock wave*[Tiab] OR extracorporeal shockwave*[Tiab] OR "Lithotripsy"[MeSH Terms] OR lithotripsy[Tiab] OR shockwave*[Tiab] OR high energy shockwave*[Tiab] OR "High-energy shock waves"[MeSH Terms] OR shock wave*[Tiab] OR radial pressure wave*[Tiab] OR acoustic wave*[Tiab] OR ESWT[Tiab] OR HESW[Tiab] OR SWT[Tiab] OR SW[Tiab] OR RPWT[Tiab] OR AWT[Tiab]) AND (Mechanotransduction [Tiab] OR Mechanotransduction, cellular[MeSH Terms] OR Mechanoreceptors[MeSH Terms] OR mechanotransduction, cellular[Tiab] OR Mechanoreceptors[Tiab] OR Mechanical signal transduction[Tiab] OR Mechanosensory transduction[Tiab] OR Target cell[Tiab] OR Receptor, cell surface[Tiab] OR Receptors, cell surface[MeSH Terms] OR Signaling pathways[Tiab] OR Signal transduction[MeSH Terms] OR Signal transduction[Tiab] OR Cell signaling[Tiab] OR Signal transduction system[Tiab] OR Receptor mediated signal transduction[Tiab] OR Signal pathways[Tiab] OR Signal transduction pathways[Tiab] OR Cellular[Tiab] OR Cells[Tiab] OR cells[MeSH Terms] OR Cell[Tiab] OR Cell physiology[Tiab] OR Cell physiological phenomena[MeSH Terms] OR Cell physiological process[Tiab] OR Cell Physiological Phenomenon[Tiab] OR Micro RNA[Tiab] OR Micro RNAs[MeSH Terms] OR Micro RNA[Tiab] OR Micro RNAs[Tiab] OR miRNA[Tiab] OR miRNAs[Tiab] OR Exosomes[MeSH Terms] OR Exosomes[Tiab])**
22/09/2022**: Results:** 1635
05/03/024: Results: 1797

Web of science

**(Fibrosis OR “pulmonary fibrosis” OR “oral submucous fibrosis” OR “endomyocardial fibrosis” OR “Cystic fibrosis” OR “connective tissue cells” OR “skin” OR “cutaneous tissue” OR “subcutaneous tissue” OR “connective tissue” OR “fibroblasts” OR “myofibroblasts” OR “tissue”) AND ("extracorporeal shockwave therapy" OR extracorporeal shock wave* OR lithotripsy OR shockwave* OR high energy shockwave* OR "High-energy shock waves" OR shock wave* OR radial pressure wave* OR acoustic wave* OR ESWT OR HESW OR SWT OR SW OR RPWT OR AWT) AND (Mechanotransduction OR Mechanotransduction, cellular OR Mechanoreceptors OR Mechanical signal transduction OR Mechanosensory transduction OR Target cell OR Receptor, cell surface OR Signaling pathways OR Signal transduction OR Cell signaling OR Signal transduction system OR Receptor mediated signal transduction OR Signal pathways OR Signal transduction pathways OR Cellular OR Cells OR cells OR Cell OR Cell physiology OR Cell physiological phenomena OR Cell physiological process OR Cell Physiological Phenomenon OR Micro RNA OR Micro RNAs OR Micro RNA OR miRNA OR miRNAs OR Exosomes)**
22/09/2022: **Results: 940**
05/03/024: Results: 945

Embase

**(Fibrosis OR “pulmonary fibrosis” OR “oral submucous fibrosis” OR “endomyocardial fibrosis” OR “Cystic fibrosis” OR “connective tissue cells” OR “skin” OR “cutaneous tissue” OR “subcutaneous tissue” OR “connective tissue” OR “fibroblasts” OR “myofibroblasts” OR “tissue”) AND ("extracorporeal shockwave therapy" OR extracorporeal shock wave* OR lithotripsy OR shockwave* OR high energy shockwave* OR "High-energy shock waves" OR shock wave* OR radial pressure wave* OR acoustic wave* OR ESWT OR HESW OR SWT OR SW OR RPWT OR AWT) AND (Mechanotransduction OR Mechanotransduction, cellular OR Mechanoreceptors OR Mechanical signal transduction OR Mechanosensory transduction OR Target cell OR Receptor, cell surface OR Signaling pathways OR Signal transduction OR Cell signaling OR Signal transduction system OR Receptor mediated signal transduction OR Signal pathways OR Signal transduction pathways OR Cellular OR Cells OR cells OR Cell OR Cell physiology OR Cell physiological phenomena OR Cell physiological process OR Cell Physiological Phenomenon OR Micro RNA OR Micro RNAs OR Micro RNA OR miRNA OR miRNAs OR Exosome)**
10/10/2022: 1312
05/03/024: 1337

Cochrane

**(Fibrosis OR “pulmonary fibrosis” OR “oral submucous fibrosis” OR “endomyocardial fibrosis” OR “Cystic fibrosis” OR “connective tissue cells” OR “skin” OR “cutaneous tissue” OR “subcutaneous tissue” OR “connective tissue” OR “fibroblasts” OR “myofibroblasts” OR “tissue”) AND ("extracorporeal shockwave therapy" OR extracorporeal shock wave* OR lithotripsy OR shockwave* OR high energy shockwave* OR "High-energy shock waves" OR shock wave* OR radial pressure wave* OR acoustic wave* OR ESWT OR HESW OR SWT OR SW OR RPWT OR AWT) AND (Mechanotransduction OR Mechanotransduction, cellular OR Mechanoreceptors OR Mechanical signal transduction OR Mechanosensory transduction OR Target cell OR Receptor, cell surface OR Signaling pathways OR Signal transduction OR Cell signaling OR Signal transduction system OR Receptor mediated signal transduction OR Signal pathways OR Signal transduction pathways OR Cellular OR Cells OR cells OR Cell OR Cell physiology OR Cell physiological phenomena OR Cell physiological process OR Cell Physiological Phenomenon OR Micro RNA OR Micro RNAs OR Micro RNA OR miRNA OR miRNAs OR Exosomes)**
10/10/2022**: 109**
05/03/024: 134

S2: Table S1. Risk of bias – detailed table

OUTCOME		STUDIES	STUDY DESIGN	ROB TOOL	1	2	3	4	5	6	7	OVERALL
Cell contraction	VEGF	[20]	Non-RCT	ROBINS-I	Low	Low	Low	Low	Low	Low	Low	**Low**
	TGFβ	[11]<br>[Yin et al., 2011] [25]<br>[Lu et al., 2020] [38]	Non-RCT	ROBINS-I	Low	Low	Low	Low	Low	Low	Low	**Low**
	Integrin	[Iannone et al., 2009] [23]<br>[Rinella et al., 2016][11]	Non-RCT	ROBINS-I	Low	Low	Low	Low	Low	Low	Low	**Low**
Fibroblasts	Fibronectin	[Cui et al., 2018][5]	Non-RCT	ROBINS-I	Low	Low	Low	Low	Low	Low	Low	**Low**
	WST-1, M-PER	[Di Stefano et al., 2020][30]	Non-RCT	ROBINS-I	Low	Low	Low	Low	Low	Low	Low	**Low**
	PCNA & fibronectin	[C. J. Wang et al., 2018][18]	Non-RCT	ROBINS-I	Low	Low	Low	Low	Low	Low	Low	**Low**
	CD117, CD90<br>PCNA<br>GF<br>Procollagen I<br>NF-kB-p65	[Di Stefano et al., 2020] [30]	Non-RCT	ROBINS-I	Low	Low	Low	Low	Low	Low	Low	**Low**
	Laminin/integrin	[Aschermann et al., 2017][3]	Non-RCT	ROBINS-I	Low	Low	Low	Low	Serious	Low	Low	**Serious**
	/	[Vetrano et al., 2019] [24]	Non-RCT	ROBINS-I	Low	Low	Low	Low	Low	Low	Low	**Low**
Myofibroblasts	CD34	[Modena et al., 2022][17]<br>[Rinella et al., 2016][11]	Non-RCT	ROBINS-I	Low	Low	Low	Low	Low	Low	Low	**Low**
	/	[Rinella et al., 2016][11]	Non-RCT	ROBINS-I	Low	Low	Low	Low	Low	Low	Low	**Low**

Fibroblasts/myofibroblasts	YAP1 protein	[Sopel et al., 2024][10]	Non-RCT	ROBINS-I	Low	Low	Low	Low	Serious	Low	Low	**Serious**
	/	[Pirri et al., 2023][27]	Non-RCT	ROBINS-I	Low	Low	Low	Low	Low	Low	Low	**Low**
αSMA	/	[Rinella et al., 2020][33]<br>[Nurzynska et al., 2008][32]	Non-RCT	ROBINS-I	Low	Low	Low	Low	Low	Low	Low	**Low**
		[Sopel et al., 2024][10]	Non-RCT	ROBINS-I	Low	Low	Low	Low	Serious	Low	Low	**Serious**
		[Leone et al., 2016][29]	Non-RCT	ROBINS-I	Low	Low	Serious	Low	Low	Some concerns	Low	**Serious**
/	CD34<br>CD44	[Saggini et al., 2015][19]	RCT	ROB-II	Low	Low	Low	Low	Low	/	/	**Low**
Fibrocytes	/	[Saggini et al., 2015][19]	RCT	ROB-II	Low	Low	Low	Low	Low	/	/	**Low**
ECM component	/	[Rinella et al., 2016][11]	Non-RCT	ROBINS-I	Low	Low	Low	Low	Low	Low	Low	**Low**
	MMP1 and 2 [remodeling and adhesion]	[Han et al., 2009][28]	Non-RCT	ROBINS-I	Low	Low	Low	Low	Low	Low	Low	**Low**
Bone marrow stromal cells	F-actin	[Suhr et al., 2013][37]	Non-RCT	ROBINS-I	Low	Low	Low	Low	Low	Low	Low	**Low**
Collagen	I<br>COL-I α1	[Rinella et al., 2016][11]<br>[C. J. Wang et al., 2018][18]<br>[Rinella et al., 2020][33]<br>[Lu et al., 2020] [38]<br>[Cui et al., 2018][5]	Non-RCT	ROBINS-I	Low	Low	Low	Low	Low	Low	Low	**Low**
		[Pirri et al., 2023][27]	Non-RCT	ROBINS-I	Low	Low	Low	Low	Low	Low	Low	**Low**
	II	[Suhr et al., 2013][37]<br>[Leone et al., 2016][29]	Non-RCT	ROBINS-I	Low	Low	Low	Low	Low	Low	Low	**Low**
	I/II	[Vetrano et al., 2019][24]	Non-RCT	ROBINS-I	Low	Low	Low	Low	Low	Low	Low	**Low**

	III	[C. J. Wang et al., 2018][18]	Non-RCT	ROBINS-I	Low	Low	Low	Low	Low	Low	Low	**Low**
	I/III	[Saggini et al., 2015][19]	RCT	ROB-II	Low	Low	Low	Low	Low	/	/	**Low**
	V	[Rinella et al., 2016][11]	Non-RCT	ROBINS-I	Low	Low	Low	Low	Low	Low	Low	**Low**
Collagen fibers	CD31	[Holsapple et al., 2021][9]	Non-RCT	ROBINS-I	Low	Low	Low	Low	Some concerns	Low	Low	**Some concerns**
		[Sopel et al., 2024][10]	Non-RCT	ROBINS-I	Low	Low	Low	Low	Serious	Low	Low	**Serious**
		[Saggini et al., 2015][19]	RCT	ROB-II	Low	Low	Low	Low	Low	/	/	**Low**
Endothelial involvement	Immunoglobulin	[Tinazzi et al., 2011] [20]	Non-RCT	ROBINS-I	Low	Low	Low	Low	Low	Low	Low	**Low**
Nitric oxide	NO	[Tinazzi et al., 2011][20]	Non-RCT	ROBINS-I	Low	Low	Low	Low	Low	Low	Low	**Low**
Cellular metabolism	cAMP	[Modena et al., 2024][12]	Non-RCT	ROBINS-I	Low	Low	Low	Low	Low	Low	Serious	**Serious**
Keratinocytes	IL-6/VEGF	[C. J. Wang et al., 2018][18]	Non-RCT	ROBINS-I	Low	Low	Low	Low	Low	Low	Low	**Low**
	Keratin 1, 6, 10, 17	[Cui et al., 2021][4]	Non-RCT	ROBINS-I	Low	Low	Low	Low	Low	Low	Low	**Low**
Epithelial cells	Keratin 5, 14	[Cui et al., 2021][4]	Non-RCT	ROBINS-I	Low	Low	Low	Low	Low	Low	Low	**Low**
Macrophages	M1-M2 macrophages<br>IL-1<br>TNF, PDGF, TGF<br>IL-6/VEGF	[Holsapple et al., 2021][9]	Non-RCT	ROBINS-I	Low	Low	Low	Low	Some concerns	Low	Low	**Some concerns**
	B-actin	[Zhai et al., 2016][26]	Non-RCT	ROBINS-I	Low	Low	Low	Low	Low	Low	Low	**Low**
Cytokines	TNFα<br>IL-6<br>IL-17A	[Vetrano et al., 2019][24]<br>[Han et al., 2009][28]	Non-RCT	ROBINS-I	Low	Low	Low	Low	Low	Low	Low	**Low**
		[Iannone et al., 2009][23]	Non-RCT	ROBINS-I	Low	Low	Low	Low	Low	Low	Low	**Low**

		[W. Wang et al., 2016][31]										
	IL-2	[Yu et al., 2023][7]	Non-RCT	ROBINS-I	Low	Low	Low	Low	Low	Low	Low	**Low**
Chemokine	ligand 1 CXCL1,2 and 3 MAPK-9	[W. Wang et al., 2016][31]	Non-RCT	ROBINS-I	Low	Low	Low	Low	Low	Low	Low	**Low**
Lymphocytes	T-lymphocytes B-lymphocytes	[Modena et al., 2022] [17]	Non-RCT	ROBINS-I	Low	Low	Low	Low	Low	Low	Low	**Low**
NK cells	/	[Modena et al., 2022] [17]	Non-RCT	ROBINS-I	Low	Low	Low	Low	Low	Low	Low	**Low**
Enzymes	COX2	[Modena et al., 2022] [17]	Non-RCT	ROBINS-I	Low	Low	Low	Low	Low	Low	Low	**Low**
Angiogenesis markers	CD14	[Rinella et al., 2016][11]	Non-RCT	ROBINS-I	Low	Low	Low	Low	Low	Low	Low	**Low**
	CD105	[Modena et al., 2022][17]<br><br>[Iannone et al., 2009] [23]	Non-RCT	ROBINS-I	Low	Low	Low	Low	Low	Low	Low	**Low**
		[Nurzynska et al., 2008][32]	Non-RCT	ROBINS-I	Low	Low	Low	Low	Low	Low	Low	**Low**
		[Modena et al., 2022] [17]<br><br>[W. Wang et al., 2016][31]<br>[Lu et al., 2020] [38]<br>[Yin et al., 2011][25]	Non-RCT	ROBINS-I	Low	Low	Low	Low	Low	Low	Low	**Low**
		[Aschermann et al., 2017][3]	Non-RCT	ROBINS-I	Low	Low	Low	Low	Serious	Low	Low	**Serious**
/	miR-19a-3p	[Gollmann-Tepekoylu et al., 2021][6]	Non-RCT	ROBINS-I	Low	Serious	Serious	Low	Low	Low	Serious	**Serious**

Proliferation	Ki67	[Vetrano et al., 2019][24] [Suhr et al., 2013][37]	Non-RCT	ROBINS-I	Low	Low	Low	Low	Low	Low	Low	**Low**
		[Leone et al., 2016][29]	Non-RCT	ROBINS-I	Low	Low	Serious	Low	Low	Some con-cerns	Low	**Serious**
		[Sopel et al., 2024][10]	Non-RCT	ROBINS-I	Low	Low	Low	Low	Serious	Low	Low	**Serious**
		[Weihs et al., 2014][8]	Non-RCT	ROBINS-I	Low	Low	Low	Low	Low	Low	Low	**Low**
Enzyme	Protein kinase	[Weihs et al., 2014] [8]	Non-RCT	ROBINS-I	Low	Low	Low	Low	Low	Low	Low	**Low**
	Protein kinase: MAP kinase	[Cui et al., 2021][4]	Non-RCT	ROBINS-I	Low	Low	Low	Low	Low	Low	Low	**Low**
/	P44/42 MAPK	[W. Wang et al., 2016][31]	Non-RCT	ROBINS-I	Low	Low	Low	Low	Low	Low	Low	**Low**
/	Total Mek 1/2	[Weihs et al., 2014][8]	Non-RCT	ROBINS-I	Low	Low	Low	Low	Low	Low	Low	**Low**
Cell surface receptors	p21, p27 and Notch1 p16	[Vetrano et al., 2019][24]	Non-RCT	ROBINS-I	Low	Low	Low	Low	Low	Low	Low	**Low**
		[Cui et al., 2021][4]	Non-RCT	ROBINS-I	Low	Low	Low	Low	Low	Low	Low	**Low**
Isoenzymes	Alkaline phosphatase	[Zhai et al., 2016][26]	Non-RCT	ROBINS-I	Low	Low	Low	Low	Low	Low	Low	**Low**
/	GADPH	[Zhai et al., 2016][26]	Non-RCT	ROBINS-I	Low	Low	Low	Low	Low	Low	Low	**Low**
T-cell	Through ATP, P2X7 receptors FAK activation and MAPK	[Yu et al., 2023][7]	Non-RCT	ROBINS-I	Low	Low	Low	Low	Low	Low	Low	**Low**
/	α/βMHC, FVIII and GADPH	[Nurzynska et al., 2008][32]	Non-RCT	ROBINS-I	Low	Low	Low	Low	Low	Low	Low	**Low**
/	Anti-MFN1 Anti-MFN2	[Modena et al., 2024]**[12]**	Non-RCT	ROBINS-I	Low	Low	Low	Low	Low	Low	Serious	**Serious**

/	miR-138 differentiation: FAK/Erk ½ pathway RUNX2	[Hu et al., 2016][21]	Non-RCT	ROBINS-I	Low	Low	Low	Low	Low	Low	Low	**Low**
/	Cbfα1	[Zhai et al., 2016][26]	Non-RCT	ROBINS-I	Low	Low	Low	Low	Low	Low	Low	**Low**
Apoptotic cells	Erk Akt signaling pAKT/totalAKT	[Holsapple et al., 2021][9]	Non-RCT	ROBINS-I	Low	Low	Low	Low	Some concerns	Low	Low	**Some concerns**
	pSTAT3/totalSTAT3	[C. J. Wang et al., 2018][18]	Non-RCT	ROBINS-I	Low	Low	Low	Low	Low	Low	Low	**Low**
	Caspase 3 Caspase 14	[Suhr et al., 2013][37]	Non-RCT	ROBINS-I	Low	Low	Low	Low	Low	Low	Low	**Low**
		[Cui et al., 2021][4]	Non-RCT	ROBINS-I	Low	Low	Low	Low	Low	Low	Low	**Low**
